# Assessment of the implementation of the Baby-Friendly Hospital Initiative in Greece

**DOI:** 10.18332/ejm/114936

**Published:** 2019-12-19

**Authors:** Evangelia Voziki, Dimitrios G. Goulis, Theofanis Vavilis, Fotios Vouzas, Maria Bouroutzoglou

**Affiliations:** 1 Papageorgiou General Hospital, Thessaloniki, Greece; 2 First Department of Obstetrics and Gynaecology, Medical School, Aristotle University of Thessaloniki, Thessaloniki, Greece; 3 Reproductive Endocrinology Unit, First Department of Obstetrics and Gynaecology, Medical School , Aristotle University of Thessaloniki, Greece; 4 Biology Department, Aristotle University of Thessaloniki, Thessaloniki, Greece; 5 Department of Business Administration, University of Macedonia, Thessaloniki, Greece; 6 Prince Mohammad Bin Fahd Univertsity, College of Business Administration, Dhahran, Saudi Arabia; 7 Department of Midwifery, School of Health Sciences, International Hellenic University, Thessaloniki, Greece

**Keywords:** breastfeeding, baby-friendly hospital, breastfeeding implementation

## Abstract

**INTRODUCTION:**

This report aims to outline the views of health professionals, working in maternity clinics, on the usefulness, benefits and difficulties of integrating the Baby-Friendly Hospital Initiative (BFHI), and to illustrate their degree of awareness about it and extent to which they are informed about BHFI.

**METHODS:**

The method of qualitative research was used to conduct the study using an open-ended questionnaire and interview technique. Sixteen health professionals working in 4 hospitals and maternity clinics in Thessaloniki (Ippokrateio, Papageorgiou, Diabalkaniko, and Genesis) participated in the interviews with equal participation of individuals in the sample.

**RESULTS:**

The extent to which respondents are informed about the Βaby-Friendly Hospital Initiative is relatively satisfactory, but there is no comprehensive knowledge of the steps required by the Harmonisation Directive. Harmonisation with the guidelines proposed by the BFHI is expected to improve the quality of obstetric clinic services and lead to a reduction in costs. However, there are particular difficulties in adapting hospitals to baby-friendly practices, particularly concerning the resources required.

**CONCLUSIONS:**

Hospitals need to make significant changes in organisation, resource efficiency and utilization, and staff training, to fully integrate the BFHI, as integration of procedures is very limited and no research has been conducted in this area in Greece.

## INTRODUCTION

The feeding practices of infants have a strong impact on the development of children under the age of two years as well as on the reduction of the risk of infectious diseases and the mortality of infants. In this sense, WHO suggests that the appropriate starting time of breastfeeding for the newborns is within an hour after the delivery and it should be followed for the first six months, supported by safe complementary nutritional supplements and continued breastfeeding until the age of two years^1^. The evidence-based guidelines of the WHO show that the application of the best breastfeeding practices are strongly related to lower risk of gastrointestinal and respiratory tract infections^2^. Moreover, breastfeeding may protect the infant from otitis^3^, malignant^4^ dental caries^5^, obesity, and type 2 diabetes^6^; it is also related to improved mental development^7^. The benefits for mothers include prolonged breastfeeding amenorrhea, and reduced risk of haemorrhage after the delivery, breast cancer, ovarian cancer and type 2 diabetes^8^.

In 1991, a joint collaboration of the international organizations WHO and UNICEF was launched in order to promote a global strategy for infant and young feeding guidelines^9^. The Baby-Friendly Hospital Initiative (BFHI) is based on a 10-step guidance to increase support for successful breastfeeding^10^ and on the WHO 1981 Code concerning the relevant aspects of the International Code of Marketing of Breastmilk Substitutes^11^. The implementation of this 10-step guidance is an evaluation directive and improvement of the quality, based on certain practices on the five sectors of breastfeeding^9,10^.

This guidance is based on four primary domains which include: a) Human Resource Development to promote the education of the hospital and obstetric clinic staff on breastfeeding policy; b) Promotion and Support, which concerns nursing prenatal education, hospital support issues, including the early start of motherly breastfeeding and support of breastfeeding groups; c) Protection, which refers to the avoidance of feeding solely through infant formulas and promotes abstinence from baby pacifiers; and d) Practice, where rooming-in allows mothers and infant to remain together for 24 hours per day throughout the hospitalization.

This study aims to identify the attitudes of health professionals regarding the implementation and communication of the practices anticipated for the certification process of the Baby-Friendly Hospital. It also aims to contribute, through the assessment of personnel attitudes and perceptions, to the better understanding and improvement of the procedures required for the implementation of the BFHI in Greece.

## METHODS

This was a mixed-methods study performed in 2015. In order to address the specific objective, a qualitative survey was conducted incorporating, where appropriate, quantitative data of the demographic and occupational characteristics of the participants included in the study. Focus-group interviews were applied as a technique for exploring complexity and diversity of participant views. Face-toface focus-group discussion was guided by fourteen fixed questions. Audiotape recordings were transcribed verbatim and later verified and validated by each participant. The processing and presentation of the data were carried out with the content analysis method appropriate for research and analysis of social communication and its proposals and consequences^12^.

The sample of the survey comprised 11 healthcare professionals (midwives and obstetricians) working in four hospitals (2 public and 2 private) of Thessaloniki; specifically, the Hippokration General Hospital, Papageorgiou General Hospital, Interbalkan Medical Centre, and Genesis Hospital. From each hospital, four people were selected to be interviewed. Nine women and seven men participated in the survey (mean age 46.4 years) with a median time of 16.8 years working in the health sector. All participants in the survey were of tertiary level education. Eight were working on a private contract while the remaining worked in the public health sector.

The survey was conducted using an open-ended questionnaire^13^ and direct interviewing. The individual interviews took place in the form of private meetings with the trainees in their workplace. The average time of an interview was about 20 minutes. The questions were asked in the same order for all participants in the survey and, where appropriate, clarifications were given.

For deeper understanding of the perceptions and attitudes of the health professionals as to the procedure but also the importance of certification of the BFHI, four primary domains were assessed: 1) The degree of education of health professionals about the BFHI and the degree of confirmation of the hospitals; 2) The benefit of the hospital’s conformation in the implementation of baby-friendly practices; 3) The benefits for the hospital, mothers and newborns through this baby-friendly procedure confirmation; and 4) The main difficulties that arise from the BFHI procedure and the implementation practices.

## RESULTS

With regard to the assessment of the degree of education of health professionals, it was determined that the degree of education of the participants about the BFHI was satisfactory. Moreover, all the participants focused particularly on the issue of exclusive direct breastfeeding and acknowledged the importance of the 24-h contact between the newborn and the mother without necessarily being aware of the steps required by the harmonisation procedure of the BFH Initiative. The degree of conformity of hospitals to this initiative was low, as the information policy on breastfeeding and aid implemented by hospitals was not organised or targeted, despite efforts of raising awareness among the doctors by the administrations. At the same time, the hospitals in Northern Greece have not developed a written policy protocol that incorporates the Ten Steps certification from the BFHI.

It is worth mentioning that in order to support and integrate the processes of the BFHI, we rely on the willingness of the staff and the administration. In cases where there is awareness among the employees outside of the obstetric clinics, positive results are observed in this specific direction. With regard to the second domain, the operation of the hospitals based on a specific protocol, and given the fact that they are friendly to infants, is considered to increase the demands of accountability and responsibility of the employees with an immediate positive impact on the quality of the services provided. Additionally, it is considered that the practice of the BFHI procedures will alleviate the burden on midwives because their presence will not be needed for the breastfeeding of newborns in Northern Greece.

Regarding the benefits for the hospital, the mothers, and the babies, the projected benefits related to an increase in the satisfaction level of the experience of the delivery action due to the continued contact with the infant, which reinforces the bonding between mother and baby, will be of great advantage also for the infant itself. As for the benefits of the implementation of the BFHI practices at the hospital level, the respondents focused on the benefits to the economic logistics of the hospitals, given the reduction in the expenditure of breastmilk substitutes. At the same time, important opportunities were also noted to arise in terms of highlighting hospitals as points of reference in society and in the medical scientific community. Besides, the benefits that arise concerning the society and the health of the newborns have an immediate impact on the hospitals in turn. Finally, it is important to stress the change in the attitude of the health professionals to provide wholesome care to mothers and newborns.

Our qualitative assessment also identified barriers to the implementation of the BFHI that include the difficulties that arise in the process of adapting hospital practices towards the BFHI procedures, which include the lack of the necessary infrastructure and the need for theoretical and practical training of the health professionals. In order for the BFHI to be effective, it should be incorporated in a broader set of overall strategies encompassing both health professionals and the hospital business culture, moving on to a hospital-centred approach rather than a medical-centred approach. Obstacles to the above are the bureaucratic nature of the system and the lack of resources and infrastructure. Specifically, the impact of the lack of resources and infrastructure on the adaptation of the BFHI was noted within the context of a broader difficulty in the implementation of ‘skin-to-skin’ deliveries because of the large percentage of caesarean sections in Greece as well as the limited ability for rooming-in.

It is worth mentioning that the focus group highlighted that difficulties in private hospitals are potentially fewer, as there is better cooperation between the staff and the administration due to a higher level of organization and the minimizing of bureaucracy issues.

## DISCUSSION

The present study highlights aspects that could lead to the implementation of the BFHI in Northern Greece as an effective method to increase the practice of exclusive breastfeeding. However, significant changes are required in hospitals, in organizational practices, in the availability of sufficient resources and their good exploitation, in staff training and generally the organizational culture of hospitals and clinics^14^. However, in Greece the number of certified BFHIs was small in 2015, when the survey took place, just five (three public and two private hospitals) in Athens out of a total of 50 private and 60 public maternity units^15^. The above are impacted on by the inadequate knowledge of staff on issues related to direct exclusive breastfeeding, the implementation of out-of-date practices, and the lack of necessary resources. The implementation of the BFHI would lead to improved satisfaction that the mothers can derive from the prenatal and neonatal care they receive. Therefore, it is necessary to establish strong administrative support, but also actions need to be taken by the medical and nursing community to incorporate the corresponding practices.

### Limitations and strengths

This study is limited by its small sample size and its focus on hospitals in Northern Greece only, and hence conclusions may not be generalizable to the entire hospital staff. However, this qualitative assessment provides some interesting initial points for the further assessment of the potential benefits and challenges of the implementation of the BFHI in Greece.

## CONCLUSIONS

It is commonly accepted that direct exclusive breastfeeding is the preferable feeding practice for the infants. Health professionals should promote and support breastfeeding, to ensure that the ideal kind of feeding is offered to all infants. In order to achieve this, breastfeeding should be supported and promoted actively by the medical community and by society. The incorporation of guidelines of the BFHI often involves resistance from the staff and doctors, because it requires the modification of traditional concepts of postnatal care, while at the same time requires that the health professionals train mothers in the skill of breastfeeding. There is also the need to support practices such as skin-to-skin contact after the birth^16^. Overall, a more wholesome approach and substantial change in the culture of society is required over this issue.
